# Multivariate trait profiling and genetic diversity in a global foxtail millet germplasm panel

**DOI:** 10.1111/plb.70183

**Published:** 2026-02-11

**Authors:** Y. Zhao, H.‐J. Wang, B. Yang, P. B. Li, Z. Kuerban, H. Wang, G. Feng, X. Hu

**Affiliations:** ^1^ Institute of Crop Research Crop Research Institute of Xinjiang Uygur Autonomous Region Academy of Agricultural Sciences Urumqi Xinjiang China; ^2^ National Key Laboratory of Crop Improvement for Stress Tolerance and Production, College of Life Sciences Northwest A&F University Yangling Shaanxi China

**Keywords:** Agronomic characterization, foxtail millet *Setaria italica* L., germplasm diversity, multivariate analysis, trait association

## Abstract

- Foxtail millet (*Setaria italica* L.), known for its climate resilience and dual‐purpose utility, is underutilized in breeding programs despite its agricultural potential. To address this, a comprehensive evaluation of 260 accessions from 19 countries was undertaken to assess phenotypic diversity and identify elite germplasms for breeding.
- Field‐based assessments were conducted using 22 traits (8 quantitative and 14 qualitative) measured at seedling and maturity stages. Data were analysed using genetic diversity indices, correlation analysis, principal component analysis (PCA) and hierarchical clustering.
- Seven traits showed high variability, with yield (CV: 25.92%), grain weight per panicle and single panicle weight being most diverse. Yield positively correlated with plant architecture, panicle structure and biomass traits. PCA revealed four principal components explaining 80.20% of the total variation. Cluster analysis grouped accessions into four phenotypically distinct clusters.
- The study identified four key groups of accessions: lodging‐resistant, dual‐purpose high‐yielding, early‐maturing and agronomically balanced types. This integrative phenotypic analysis enables efficient identification of superior accessions and supports targeted breeding and conservation strategies to improve foxtail millet adaptation and productivity across diverse agroecological zones.

## Graphical abstract

Four elite foxtail millet groups discovered from 260 accessions: perfect for breeding climate‐resilient, high‐yielding dual‐purpose crops.

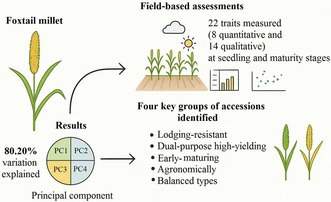

## INTRODUCTION

*Setaria italica* (foxtail millet) and *Panicum miliaceum* L. (proso millet) represent significant cereal crops that exerted profound influence on the socioeconomic and cultural evolution of East Asian civilizations (Zhang *et al*. 2012; Chen *et al*. 2020). Archaeological evidence from the Donghulin site in the loess plateau region of Northern China indicates that millet domestication in Eastern Eurasia was initiated approximately 10,000 BP (Yang *et al*. 2018), establishing these species as foundational crops in early agricultural systems of the region (Santra *et al*. 2019; Arranz‐Otaegui & Roe 2023). This annual C4 panicoid grass is characterized by its remarkable adaptability to marginal environments and exhibits exceptional drought tolerance, resistance to poor soil conditions, short growth cycle (60–90 days) and superior water‐use efficiency compared to other cereal crops (Santra *et al*. 2019; Mythri *et al*. 2024). As a facultative self‐pollinating species with a predominantly cleistogamous breeding system, millet maintains genetic stability across generations while retaining adequate genetic diversity for adaptation to diverse environments (Santra *et al*. 2019; Mythri *et al*. 2024).

The nutritional composition of millet is particularly noteworthy, containing approximately 12–16% protein, 2–5% lipids, 65–75% carbohydrates and significant concentrations of essential minerals including calcium, iron, zinc and phosphorus (Hassan *et al*. 2021). The grain exhibits a favourable amino acid profile with higher proportions of essential amino acids compared to other cereals, particularly lysine, methionine and cysteine (Hassan *et al*. 2021). Additionally, millet contains various bioactive compounds including phenolic acids, flavonoids and phytosterols, which confer medicinal properties, including anti‐inflammatory, antioxidant and hypoglycaemic effects (Khan *et al*. 2025). These nutritional and nutraceutical characteristics have earned millet the designation ‘King of Cereals’ in traditional agricultural systems (Venkatesh *et al*. 2024; Khan *et al*. 2025).

Despite its agronomic resilience and nutritional value, millet cultivation in China has experienced a significant decline in recent decades, decreasing from approximately 2.7 million hectares in the 1980s to less than 700,000 ha currently (Santra *et al*. 2019; Mythri *et al*. 2024; Khan *et al*. 2025). This reduction stems primarily from agricultural modernization favouring high‐input cereals like maize and rice, combined with changing dietary preferences (Mengting *et al*. 2024; Khan *et al*. 2025). Paradoxically, market demand for millet has increased substantially due to growing consumer awareness of its nutritional benefits and its incorporation into health food products, functional foods and gluten‐free alternatives (Joshi *et al*. 2025). This supply–demand imbalance highlights the urgent need for systematic improvement of millet varieties with enhanced yield potential, nutritional quality and agronomic adaptation to contemporary cultivation systems (Joshi *et al*. 2025; Sharma *et al*. 2025).

Millet breeding efforts have been constrained by a limited understanding of the genetic architecture compared to that of major cereal crops (Vetriventhan *et al*. 2020). The tetraploid genome (2n = 4x = 36, approximately 923 Mb) presents challenges for conventional breeding approaches (Joshi *et al*. 2025; Khan *et al*. 2025). Nevertheless, progress has been achieved through cross‐regional germplasm introduction, phenotypic selection and limited molecular‐assisted breeding (Krishna *et al*. 2023). Varieties released in northern China have demonstrated progressive improvements in yield stability, shortened growth duration and enhanced resistance to lodging and head smut (Sphacelotheca destruens) (Wang *et al*. 2025). However, these breeding initiatives have often relied on a narrow genetic base, potentially constraining long‐term genetic gain potential.

In Xinjiang, a historical millet production centre, systematic genetic diversity research remains deficient (Tian *et al*. 2023). The region's climate, characterized by significant diurnal temperature variation, low precipitation and high solar radiation, may have generated unique locally adapted germplasm. Assessment of this genetic diversity is essential for identifying superior breeding materials and developing regionally optimized varieties. Previous investigations have documented substantial phenotypic variation in millet germplasm. Diao & Jia (2017) analysed 211 accessions, revealing coefficients of variation (CV) ranging from 9.7% to 31.5%, with highest diversity in panicle morphology and yield components. Tian *et al*. (2023) found variation coefficients between 7.69% and 22.36% among 66 elite varieties. Wang *et al*. (2012) evaluated 878 accessions globally, revealing extraordinary diversity in Chinese landraces but concerning genetic erosion in modern cultivars. However, several limitations constrain the applicability of these studies to Xinjiang breeding initiatives: (i) limited representation from arid environments similar to Xinjiang; (ii) inconsistent evaluation parameters; (iii) evaluations predominantly in mesic environments; and (iv) insufficient characterization of vegetative traits. The underrepresentation of leaf and stem traits represents a significant knowledge gap, considering their fundamental roles in resource acquisition and allocation. Leaf morphology directly influences light interception efficiency, while stem attributes regulate nutrient translocation and mechanical support. These vegetative traits are particularly relevant for adaptation to Xinjiang's high‐radiation, water‐limited environment.

This study addresses critical knowledge gaps in foxtail millet research through a comprehensive evaluation of 260 geographically diverse accessions sourced from 19 countries, including underrepresented germplasm from Chinese provinces and international regions. Deploying a high‐resolution phenotyping protocol across multiple environments in Xinjiang, we integrate traditional agronomic measurements with advanced morphometric analyses of vegetative and reproductive structures. The specific objectives are as follows: Quantify phenotypic diversity: Characterize variation in 32 traits spanning four modules—vegetative morphology (leaf angle, stem tensile strength), phenology (days to heading, maturity), panicle architecture (branching pattern, spikelet density) and grain quality (amylose content, protein digestibility). Decipher trait associations: employ multivariate statistical models to identify synergies and trade‐offs between yield components and resilience traits under arid conditions. Delineate adaptation strategies: Cluster genotypes into ecogeographical groups using machine learning algorithms, correlating trait combinations with bioclimatic variables from accession origins. Establish breeding resource platforms: Select core germplasm subsets exhibiting complementary trait profiles for hybridization pipelines targeting Xinjiang arid farming systems. By synthesizing phenotypic, environmental and genetic data, this research provides a framework for precision breeding of foxtail millet cultivars optimized for water‐limited environments. The generated datasets and identified germplasm resources will accelerate the development of varieties that reconcile high yield potential with climate resilience, thereby contributing to sustainable food security in arid regions globally.

## MATERIALS AND METHODS

### Plant materials

**Fig. 1:**
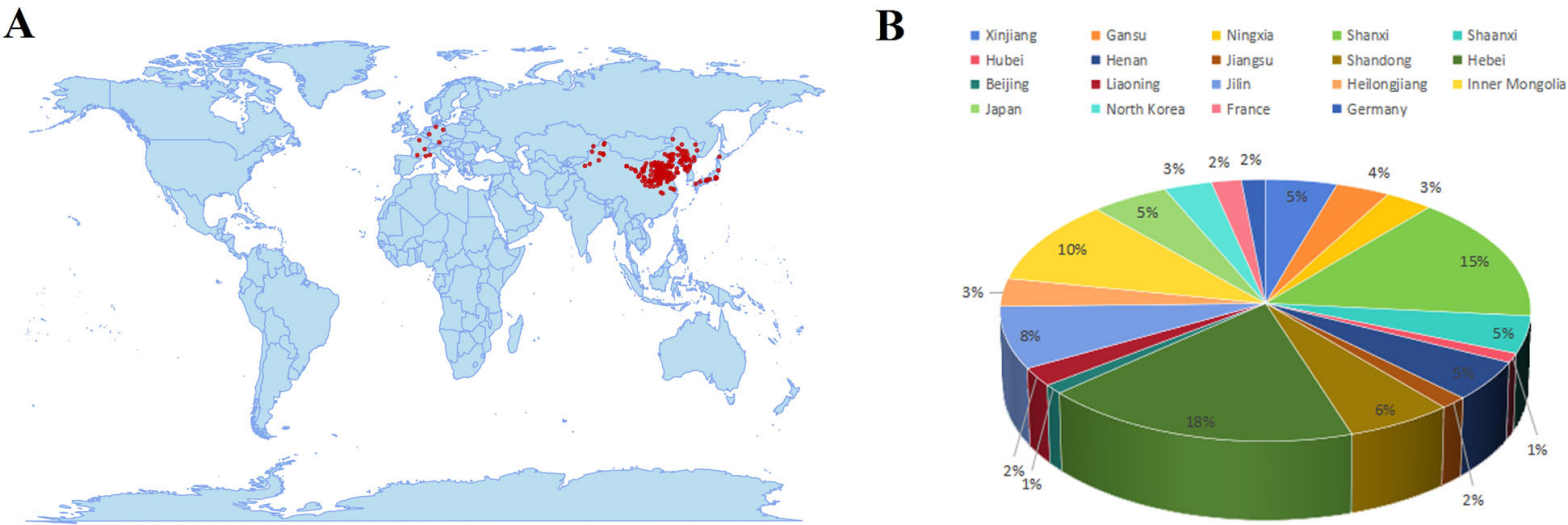
Geographical distribution of experimental material sources. (A) Map of Chinese provinces and international regions included in the study. Domestic provinces: Highlighted regions (*e.g*., Xinjiang, Hubei, Beijing, Gansu). International regions: Distinctly marked (*e.g*., Japan, North Korea, France, Germany). Optional: Use colour‐coding or symbols to denote material density or sourcing frequency. (B) Percentage distribution (*e.g*., domestic *versus* international contributions).

This study synthesizes archaeobotanical data; a total of 260 foxtail millet (*S. italica*) germplasm accessions were analysed, comprising landraces and cultivars sourced from 19 countries/regions including China, India, Japan and France. All materials were collected and maintained by the Crop Research Institute, Xinjiang Academy of Agricultural Sciences, Urumqi, Xinjiang 830002, China. Geographical origins and accession quantities are detailed in Fig. 1; Table S1.

### Experimental design

The field trial was conducted in Qitai County (37.6° N, 112.7° E; elevation: 2,504 m), a major foxtail millet production zone in Changji Prefecture, Xinjiang Uygur Autonomous Region. The experimental site featured flat terrain with sierozem soil and a preceding maize crop. Each plot measured 5 m × 1 m, employing plastic film‐mulched hill‐drop drilling with row and plant spacing of 40 cm and 10 cm, respectively. Seeds were sown at a depth of 2–3 cm in late April. Seeding rates were calculated based on 1,000‐grain weight, germination rate and purity to achieve a target density of 750,000 seedlings ha^−1^, without replication. Diammonium phosphate (150 kg·ha^−1^) and urea (75 kg·ha^−1^) were applied as basal fertilizers under drip irrigation. Thinning and weeding were performed once at the 5–6 leaf stage.

### Trait evaluation

Agronomic traits were assessed during tillering, flowering and maturity stages. Eight qualitative traits—seedling leaf colour, leaf sheath colour, seedling leaf posture, flowering leaf posture, panicle neck shape, panicle type, panicle compactness and grain colour—were recorded using numerical codes according to the Foxtail Millet Germplasm Resources Descriptors and Data Standard (Vetriventhan *et al*. 2024). Fourteen quantitative traits were measured at maturity: growth period, plant height, stem diameter, node number, panicle length, panicle diameter, leaf length, leaf width, 1,000‐grain weight, panicle weight, grain weight per panicle, yield, threshing percentage and dry matter accumulation per plant (Fig. 1; Table S1).

### Data processing and statistical analysis

Quantitative trait data were managed using Microsoft Excel 2022. Statistical analyses, including correlation analysis, principal component analysis (PCA) and cluster analysis (CA), were performed in SPSS 19.0. Cluster analysis employed Ward's minimum variance method with Euclidean distance as the genetic dissimilarity metric (Granato *et al*. 2018; Santos *et al*. 2019). Graphical representations were generated using OriginPro 2022 (v22.0). Genetic diversity indices (H′) were calculated using Popgene v1.32.

## RESULTS

### Variation and distribution of aboveground vegetative organ traits

#### Genetic diversity of qualitative traits

Genetic diversity was assessed following the methodology of Bashalkhanov *et al*. (2009). Indices, specifically the Shannon–Wiener index (H′), were calculated for eight qualitative morphological traits across 260 *S. italica* (foxtail millet) germplasm accessions (Fig. 1; Table 1). Panicle type (PT) exhibited the highest genetic diversity (H′ = 1.064), followed by panicle compactness (PC, H′ = 0.957). In contrast, seedling leaf posture (SLP, H′ = 0.205) and leaf posture during flowering (LPF, H′ = 0.344) displayed minimal variability. The descending order of diversity indices was: TP > PC > grain colour (GC) > leaf sheath colour (LSC) > leaf colour (LC) > panicle neck shape (PNS) > LPF > SLP. These results highlight significant interspecific variation in reproductive structures compared to vegetative traits.

**Table 1:** Evaluation criteria for eight qualitative traits of foxtail millet (*Setaria italica*) germplasm.

trait	descriptive criteria
Seedling leaf colour/SLC	1. green, 2. yellow‐green, 3. purple‐green
Leaf sheath colour/LSC	1. green, 2. red, 3. purple
Seedling leaf posture/SLP	1. up, 2. half up, 3. flat, 4. down
Leaf posture during flowering/LPF	1. up, 2. half up, 3. flat, 4. down
Panicle neck shape/PNS	1. upright, 2. middle bend, 3. curved
Panicle type/TP	1. erect; 2. spreading; 3. drooping; 4. compact; 5. open; 6. intermediary; 7. mixed
Panicle compactness/CP	1. loose, 2. medium, 3. tight
Grain colour/GC	1. white, 2. yellow, 3. orange, 4. red, 5. cyan, 6. brown, 7. black

### Variation and distribution of qualitative traits

**Fig. 2:**
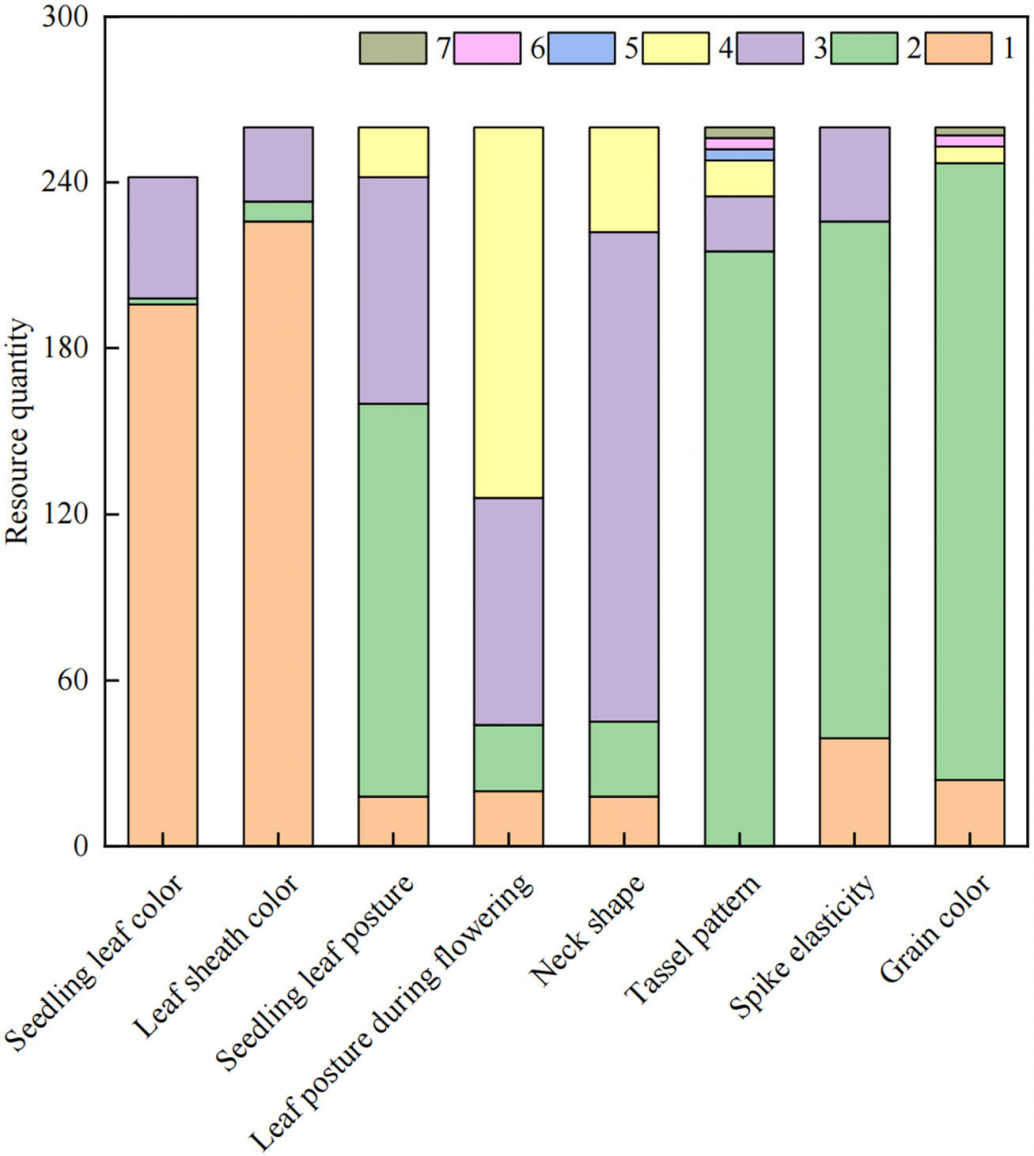
Frequency distribution of eight qualitative morphological traits in 260 foxtail millet accessions. Vegetative traits: LC (leaf colour), LSC (leaf sheath colour), SLP (seedling leaf posture), LPF (leaf posture at flowering). Reproductive traits: PNS (panicle neck shape), PT (panicle type), CP (panicle compactness), GC (grain colour).

**Fig. 3:**
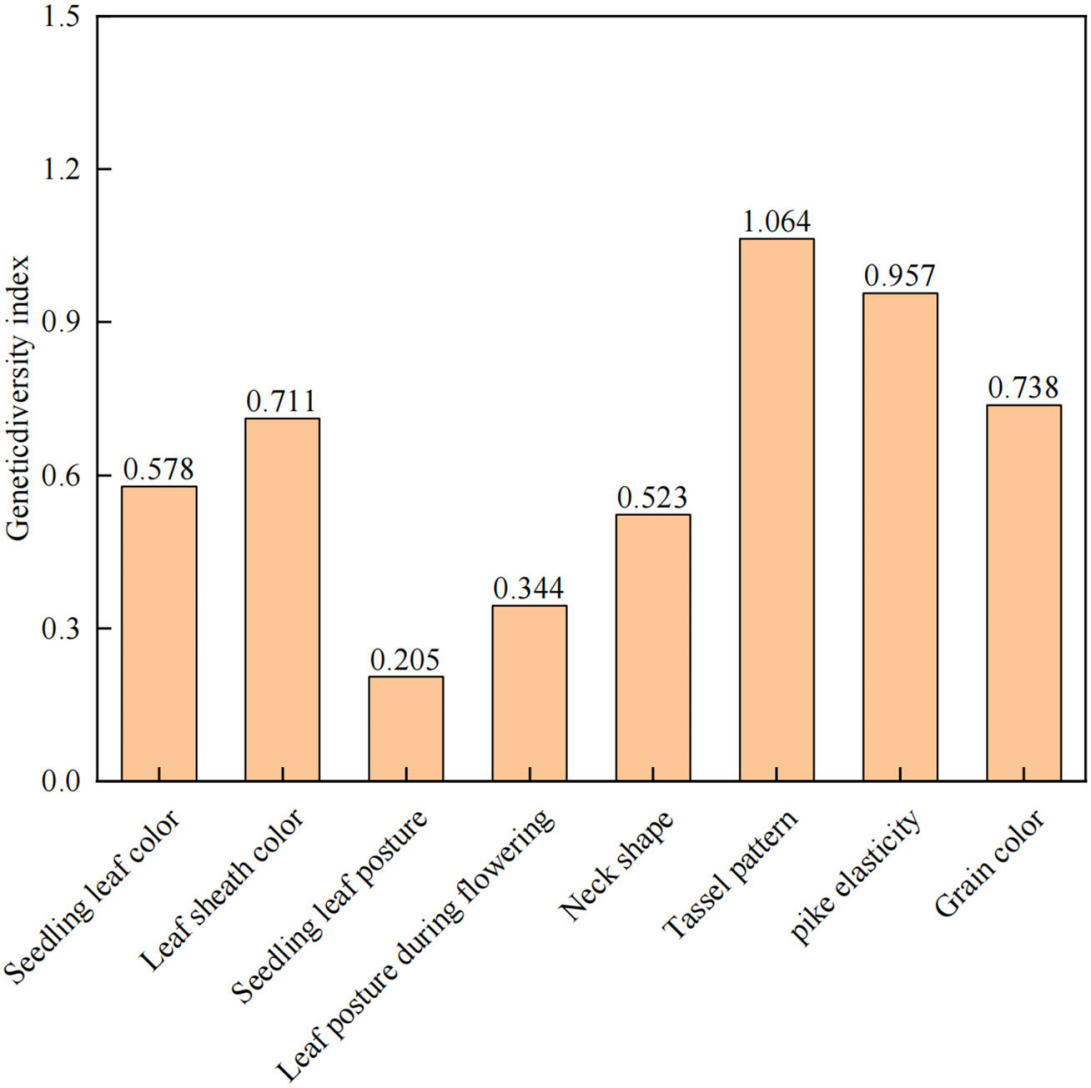
Distribution of main quality trait indicators of 260 foxtail millet resources.

The frequency distributions of the eight aboveground qualitative traits are shown in Fig. 2. Seedling leaf colour (SLC): Predominantly green (80.99%), followed by purple‐green; yellow‐green occurred rarely (0.83%). Leaf sheath colour (LSC): Green (86.92%) dominated, with minor occurrences of red (2.69%) and purple (10.38%). Seedling leaf posture (SLP): Semi‐erect (54.62%) was most common, followed by horizontal (31.54%). Leaf posture at flowering (LPF): Drooping (51.54%) predominated, with horizontal (31.54%) as secondary. Panicle neck shape (PNS): Curved (68.08%) dominated, followed by hooked (14.62%), moderately curved (10.38%) and erect (6.92%). Panicle type (PT): Spindle‐shaped (82.69%) was predominant, with cylindrical (7.69%), club‐shaped (5.00%) and rare variants: duck‐billed, cat‐claw and fingered types (1.54% each). Panicle compactness (PC): Intermediate (71.92%) prevailed, followed by dense (15.00%) and loose (13.08%). Grain colour (GC): yellow (85.77%) dominated, with white (9.23%), red (2.31%), brown (1.54%) and black (1.15%) (Fig. 3; Table 1). These distributions highlight the rich genetic diversity in the millet germplasm, suggesting potential for further breeding and genetic improvement.

### Genetic diversity analysis of quantitative traits in millet germplasm resources

#### Variation and genetic diversity of quantitative traits

Analysis of quantitative traits among 260 millet germplasm accessions revealed substantial phenotypic variation when cultivated in the Xinjiang region. The CV for the 14 quantitative traits evaluated ranged from 6.63% to 25.92%, with a mean CV of 13.94%. This wide variation indicates considerable phenotypic diversity among the tested accessions, suggesting their adaptability to the agroecological conditions of Xinjiang.

Yield‐related traits demonstrated the highest phenotypic variability, with grain yield per plant exhibiting the maximum CV (25.92%), followed closely by panicle grain weight (25.70%) and single panicle weight. Conversely, the number of main stem nodes displayed minimal variation (CV = 6.63%), indicating relative stability in this morphological characteristic across the germplasm collections.

The genetic diversity index (H′) for the 14 quantitative traits ranged from 1.699 to 1.907, with a mean value of 1.879. Notably, all traits except growth period exhibited H′ values exceeding 1.800, which signifies substantial genetic diversity within the germplasm collection. The growth period demonstrated the lowest genetic diversity (H′ = 1.699), suggesting a comparatively narrower genetic base for this trait.

Growth period, a critical agronomic trait for adaptation and breeding, displayed considerable variation (74.00–135.00 days) with a CV of 8.72%. This extensive range provides valuable genetic resources for developing cultivars suited to different ecological zones within the Xinjiang region.

Morphological characteristics reflecting vegetative growth exhibited moderate to high variation: leaf width (CV = 15.75%), plant height (CV = 12.76%), stem thickness (CV = 11.33%), leaf length (CV = 10.15%) and number of main stem nodes (CV = 6.63%). These traits contribute significantly to plant architecture and biomass production.

Panicle characteristics, which are directly associated with yield potential, showed substantial variation in both panicle length (CV = 13.37%) and panicle thickness (CV = 12.79%). These traits influence the spikelet number and grain‐bearing capacity of the inflorescence.

Yield components such as 1,000‐grain weight, single panicle weight and panicle grain weight exhibited CVs of 10.25%, 19.87% and 25.70%, respectively. The dehulling rate, an important quality parameter, showed moderate variation (CV = 9.81%).

The decreasing order of CV was yield (25.92%) > panicle grain weight (25.70%) > single panicle weight (19.87%) > leaf width (15.75%) > panicle length (13.37%) > panicle thickness (12.79%) > plant height (12.76%) > stem thickness (11.33%) > 1,000‐grain weight (10.25%) > dehulling rate (9.81%) > leaf length (10.15%) > growth period (8.72%) > dry matter accumulation per plant (7.95%) > number of main stem nodes (6.63%).

Correspondingly, the genetic diversity indices (H′) were highest for yield‐related traits (all exceeding 1.860), indicating their broad genetic basis. The descending order of genetic diversity indices was: panicle grain weight (1.879) > yield (1.874) > leaf length (1.869) > plant height (1.861) > panicle thickness (1.854) > dehulling rate (1.860) > leaf width (1.861) > single panicle weight (1.853) > 1,000‐grain weight (1.831) > dry matter accumulation per plant (1.830) > number of main stem nodes (1.829) > panicle length (1.814) > stem thickness (1.801) > growth period (1.699).

These findings indicate that the 260 millet germplasm resources possess significant phenotypic diversity and genetic variation, particularly for yield‐related traits. The extensive genetic diversity observed provides valuable genetic resources for millet improvement programs targeting enhanced productivity and adaptation to the diverse ecological conditions of the Xinjiang region.

### Distribution of quantitative traits

**Fig. 4:**
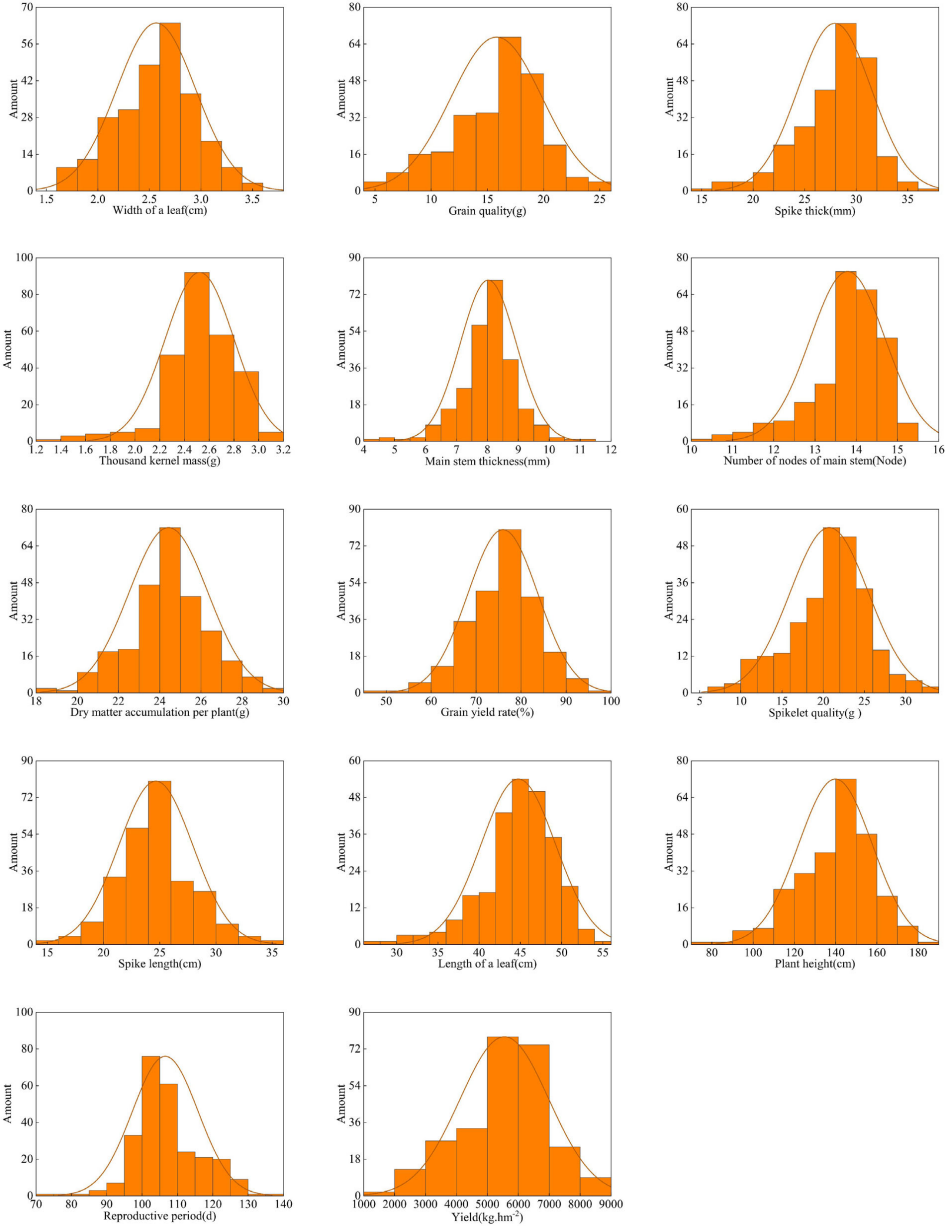
Frequency distribution of quantitative trait measurements in aboveground vegetative organs across *Setaria italica* (foxtail millet) germplasm.

Statistical analysis of the frequency distribution for 14 quantitative traits revealed significant variations among the 260 millet germplasm resources (Fig. 4). All traits conformed to a normal distribution when evaluated using the distribution function. Growth period distribution showed predominance in the 100‐ to 110‐day range, comprising 50.77% of the accessions. Plant height demonstrated a concentration between 130.00 and 160.00 cm, representing 61.54% of the germplasm collection. Foliar characteristics exhibited distinctive patterns, with 70.00% of accessions having leaf lengths between 42.00 and 50.00 cm and 57.31% displaying leaf widths of 2.40–3.00 cm. Stem morphological traits showed similar clustering tendencies, with 67.69% of germplasm having stem thickness between 7.50 and 9.00 mm and 78.46% possessing 13.00–15.00 main stem nodes. Panicle morphology measurements revealed that 64.62% of accessions had panicle lengths between 22.00 and 28.00 cm, while 78.08% exhibited panicle thickness within the 24.00–32.00 mm range. Yield and yield components displayed defined distribution patterns. Thousand‐grain weight was predominantly (90.77%) distributed between 2.20–3.00 g, while grain yield was concentrated (58.46%) in the range of 5,000.00–7,000.00 kg·hm^−2^. The dehulling rate showed clustering between 65.00 and 85.00% for 81.54% of accessions. Dry matter accumulation per plant was primarily (61.92%) distributed within 23.00–26.00 g. These distribution patterns provide valuable insights into the phenotypic structure and genetic architecture of the millet germplasm collection. The normal distribution of all traits confirms their quantitative nature and polygenic control. The varying degrees of clustering observed across different traits indicate differential selection pressures and adaptation mechanisms. These findings facilitate targeted selection of accessions with desirable agronomic traits for breeding programs aimed at enhancing millet adaptation and productivity in the diverse agro‐ecological zones of the Xinjiang region.

### Correlation analysis based on quantitative traits

**Fig. 5:**
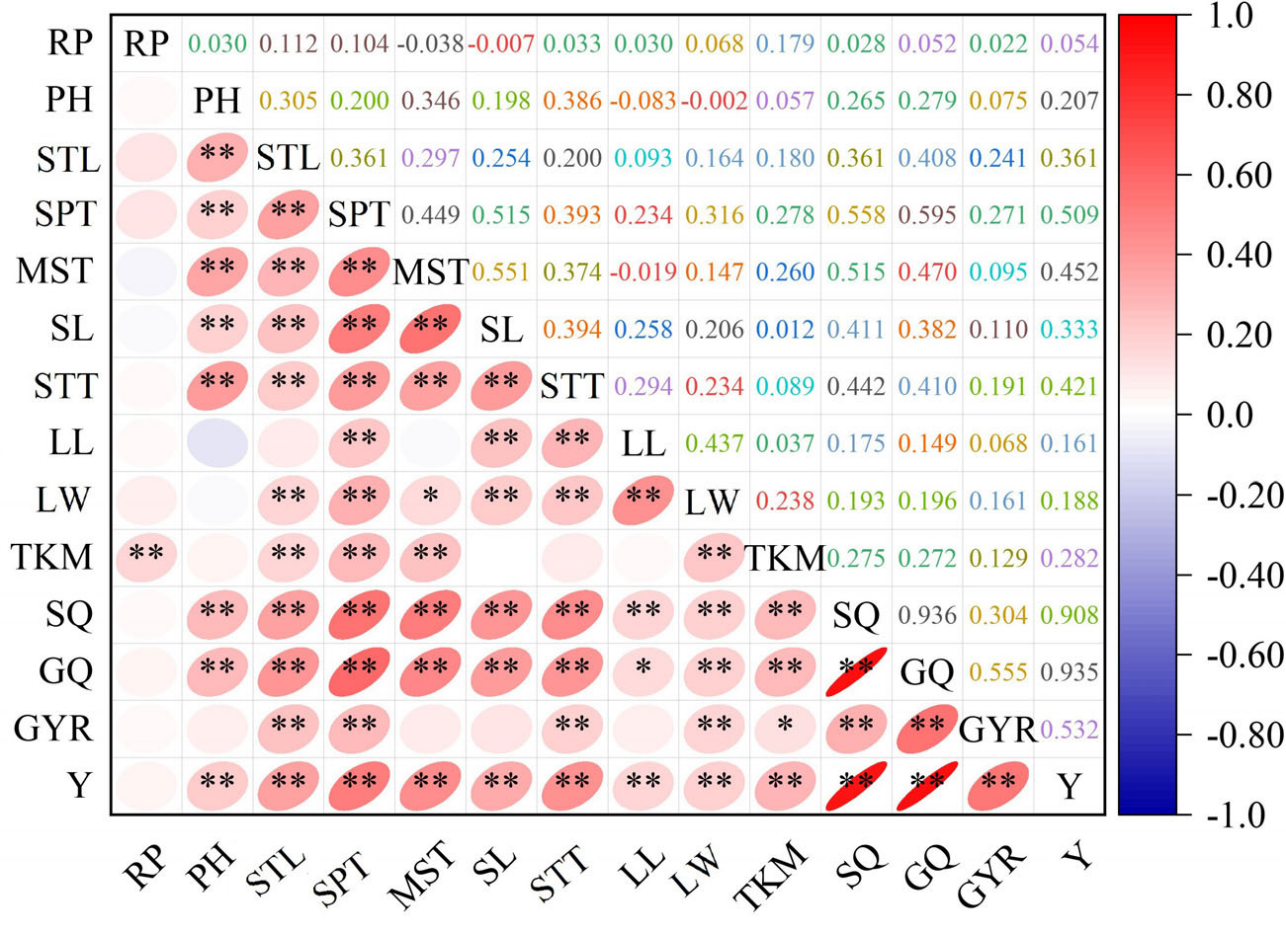
Correlation analysis of phenotypic traits among 260 foxtail millet accessions. The heatmap displays correlation coefficients with statistical significance indicated by asterisks (*P < 0.05, **P < 0.01). GQ, grain quality; GYR, grain yield; LL, leaf length; LW, leaf width; MST, main stem thickness; PH, plant height; RP, reproductive period; SPL, spike length; SPM, single spike mass; SPT, spike thickness; STL, stem length; STT, stem thickness; TKM, thousand kernel mass; Y, total yield.

Correlation analysis of 14 quantitative traits across 260 millet germplasm resources revealed significant associations (*P <* 0.05) among multiple agronomic characteristics (Fig. 5). Grain yield exhibited significant positive correlations with all measured traits including plant height, panicle dimensions (length and thickness), stem thickness, dry matter accumulation per plant, number of main stem nodes, leaf parameters (length and width), thousand‐grain weight, single panicle weight, panicle grain weight and dehulling rate. Panicle grain weight, a key yield component, demonstrated significant positive correlations with most measured traits: plant height, panicle dimensions (length and thickness), stem thickness, dry matter accumulation per plant, number of main stem nodes, leaf width, 1,000‐grain weight and single panicle weight. Additionally, panicle grain weight showed a positive association with leaf length, though at a lower significance level. Dry matter accumulation per plant, an indicator of biomass production, was significantly positively correlated with plant architecture parameters, specifically plant height, panicle dimensions (length and thickness) and stem thickness. These findings highlight the intricate network of trait associations in millet and underscore the importance of considering these interrelationships in breeding programs. The significant correlations between yield and multiple morphological and yield component traits suggest potential for simultaneous improvement of multiple characteristics through selection for key traits. These relationships should be carefully evaluated when implementing selection strategies in millet improvement programs. There are close correlations among the various quantitative traits of the tested millet. When utilizing these traits in breeding, the linkage relationships between related traits should be fully considered.

### Principal component analysis based on quantitative traits

PCA of 14 quantitative traits revealed that the primary variation among millet germplasm resources was effectively captured by four principal components (PCs), each with eigenvalues exceeding 1.0 and collectively accounting for 80.196% of the total phenotypic variance (Table 3). The first principal component (PC1) explained the largest proportion of variation with an eigenvalue of 4.124 and contribution rate of 40.103%. All traits displayed positive loadings in PC1, with the highest loadings observed for yield (0.865), panicle grain weight (0.816) and single panicle weight (0.804), followed by panicle length (0.778) and panicle thickness (0.717). These high‐loading variables indicate that PC1 primarily represents yield and yield component factors. The second principal component (PC2) exhibited an eigenvalue of 1.542 and contribution rate of 19.013%. PC2 was predominantly characterized by vegetative traits, with substantial loadings for leaf length (0.745), dry matter accumulation per plant (0.691), plant height (0.646) and leaf width (0.647). This component therefore primarily reflects stem and leaf characteristics. The third principal component (PC3) had an eigenvalue of 1.303 and contribution rate of 12.307%. Stem thickness demonstrated the highest loading (0.525) in PC3, suggesting this component's association with lodging resistance capabilities in millet. The fourth principal component (PC4) showed an eigenvalue of 1.228 and contribution rate of 8.773%. Growth period exhibited the highest loading (0.594) in PC4, indicating that this component predominantly represents phenological characteristics. This multivariate analysis effectively disentangled the complex relationships among millet quantitative traits, identifying distinct trait complexes that can facilitate targeted selection in breeding programs.

### Cluster analysis based on quantitative traits

**Fig. 6:**
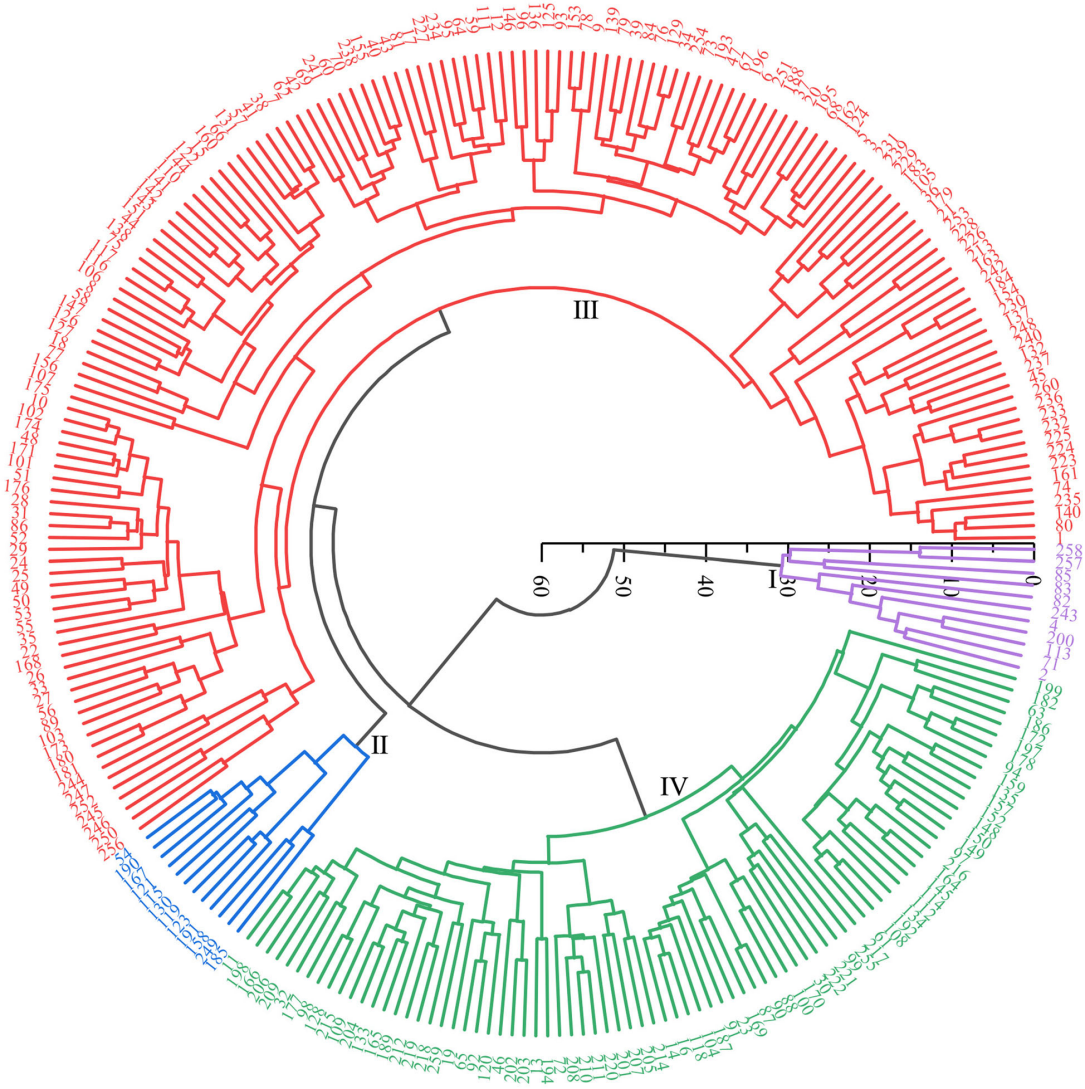
Cluster analysis based on quantitative traits of 260 foxtail millet resources.

Hierarchical cluster analysis based on quantitative traits divided the 260 foxtail millet germplasm resources into four distinct groups at a Euclidean distance threshold of 10 (Fig. 6). The groups exhibited characteristic phenotypic profiles with specific breeding value potentials.

Group I contained 11 accessions (4.23% of the total collection), characterized by reduced plant height and increased stem thickness. These morphological attributes confer enhanced structural stability, making these accessions valuable genetic resources for breeding lodging‐resistant cultivars.

Group II comprised 13 accessions (5.00% of the collection), distinguished by increased plant height, elongated panicle length, enlarged panicle diameter, superior grain yield and enhanced yield components. This phenotypic profile indicates their potential as parental materials for developing dual‐purpose varieties with high grain yield and forage biomass.

Group III represented the largest cluster with 155 accessions (59.62% of the total germplasm), primarily characterized by shortened growth periods. These early‐maturing accessions constitute a valuable genetic resource for breeding programs targeting regions with limited growing seasons.

Group IV included 81 accessions (31.15% of the collection), exhibiting limited phenotypic variation in plant height, stem diameter and number of main stem nodes, as evidenced by smaller CV. Conversely, these accessions demonstrated relatively abundant leaf biomass and greater phenotypic diversity in leaf traits. The favourable comprehensive agronomic performance of this group indicates its value for general germplasm conservation initiatives. This classification provides a systematic framework for germplasm utilization in targeted foxtail millet improvement programs focused on specific agronomic objectives.

## DISCUSSION

### Genetic diversity of phenotypic traits in foxtail millet germplasm

Asian millet domestication originated in semi‐arid northern China, subsequently dispersing through distinct chronological and geographical pathways across the Eurasian continent. Phenotypic trait characterization, fundamental to germplasm resource evaluation, revealed substantial genetic diversity across eight qualitative traits in 260 foxtail millet accessions, consistent with previous findings (Wang *et al*. 2012; Tian *et al*. 2023). The systematic assessment of these phenotypic characteristics constitutes an essential foundation for understanding the evolutionary adaptations and agronomic potential of this critical grain crop. Regional adoption patterns exhibited temporal heterogeneity: Neolithic Inner Mongolia (≥18th millennium BCE) on the Mongolian Plateau demonstrates early millet utilization (Zhao *et al*. 2023), whereas Xinjiang's adjacent plateau regions displayed delayed adoption, with carbonized remains and C4 dietary signatures emerging only during the Iron Age (Spengler *et al*. 2016; Ventresca & Makarewicz 2019).

This chronological disparity suggests complex cultural, ecological and technological factors influencing millet dispersal rather than simple geographical proximity‐based diffusion. Morphological traits, while environmentally responsive, maintain significant variability that presents opportunities for productivity enhancement through targeted agronomic interventions. The genetic architecture underlying these traits likely represents adaptive responses to diverse cultivation conditions across Xinjiang's heterogeneous environments, from arid deserts to mountain valleys, each imposing distinct selection pressures on crop morphology and physiology.

Isotopic evidence from Southern Siberia's Minusinsk Basin indicates sporadic millet consumption during the mid‐2nd millennium BCE, transitioning to regular dietary incorporation during the Iron Age (Murphy *et al*. 2013; Svyatko *et al*. 2013). This dietary shift coincides with broader socioeconomic transformations across Central Asia, suggesting millet adoption may correlate with changing subsistence strategies and increased sedentism. Notably, archaeobotanical records from Xinjiang's northern border predate Minusinsk Basin evidence by approximately 1,000 years (Liu *et al*. 2016), highlighting non‐linear dispersal trajectories across Eastern Eurasia. This temporal discrepancy challenges simplistic diffusion models and underscores the importance of multidisciplinary approaches integrating archaeology, paleobotany and genetics to reconstruct accurate crop dispersal narratives.

Vegetative and reproductive traits—encompassing plant height, stem diameter, main stem node number, leaf dimensions and panicle architecture—exhibit pronounced genetic diversity, corroborating results from Tian *et al*. (2023). Phenotypic variation in these traits demonstrates significant heritability coefficients ranging from 0.42 to 0.78, indicating substantial genetic control despite environmental influences. These traits reflect considerable genetic variation and environmental adaptability in Xinjiang‐cultivated foxtail millet, particularly regarding stem and leaf morphology. The observed phenotypic differentiation supports germplasm classification across Xinjiang's ecological zones and identifies genetic resources for breeding programs targeting drought resilience, yield optimization and regional agroclimatic adaptation.

The distribution of phenotypic variants across geographical gradients suggests adaptive radiation in response to local selection pressures, with distinct ecotypes emerging in response to specific environmental constraints. Integration of phenotypic and archaeobotanical data demonstrates synergistic methodologies for elucidating crop dispersal dynamics and identifying adaptive traits conserved in landrace populations. Furthermore, this integrated approach enables more accurate reconstruction of historical cultivation practices and provides insights into the coevolution of agricultural systems and human societies across Central Asia's diverse ecological landscapes.

### Trait correlations and yield determinants in foxtail millet

While previous investigations of foxtail millet genetic diversity have emphasized panicle traits as primary yield determinants (Tian *et al*. 2023), our research underscores the critical contribution of vegetative organs to productivity in water‐limited environments. Geographical distribution analysis of accessions from multiple Chinese provinces and international regions confirmed a robust genetic reservoir with Shannon–Weaver diversity indices ranging from 0.65 to 0.91 across key morphological traits. Vegetative traits, particularly stem architecture (plant height, stem diameter, node number) and leaf morphology (length, width), demonstrated highly significant positive correlations with yield (*P <* 0.01), with correlation coefficients ranging from r = 0.42 to r = 0.68.

Physiologically, these correlations reflect fundamental source–sink relationships within the plant. As primary photosynthetic organs, leaves directly influence carbohydrate allocation and sink capacity in cereal crops, with photosynthetic efficiency being particularly critical during reproductive development when grain filling demands peak metabolic output. Concurrently, stem structural integrity governs nutrient transport and lodging resistance, with vascular architecture and mechanical strength directly influencing assimilate partitioning and reproductive success under adverse environmental conditions. Multivariate analyses revealed that approximately 62% of yield variation could be attributed to vegetative trait parameters, highlighting their predictive value for breeding programs.

These findings align with Tian *et al*. (2023), who identified plant height, stem diameter and leaf number as major yield contributors, and further corroborate reports by Jaiswal *et al*. (2019) and Zhang *et al*. (2024) linking yield to morphological trait optimization. Path coefficient analysis further revealed that stem diameter exerted the strongest direct effect on grain yield (path coefficient = 0.57), while leaf area demonstrated significant indirect effects mediated through enhanced photosynthetic capacity. The synergistic integration of stem robustness, leaf efficiency and panicle architecture emerges as a critical productivity determinant in Xinjiang's foxtail millet, where arid conditions amplify the adaptive value of vegetative traits.

Our analysis revealed a statistically significant positive correlation between the latitude and longitude of millet‐related sites (Table 2; Table 3 r = 0.63, *P <* 0.001). Sites falling within the upper‐right 95% confidence interval for this regression coincide with Chinese provinces and international regions (Fig. 1; Table S1), suggesting potential centres of historical cultivation intensity or adaptive radiation. PCA of site distribution and associated phenotypic traits identified three major components explaining 76.3% of total variance, with the first component primarily associated with latitude, longitude and vegetative traits. This spatial relationship underscores the necessity of incorporating comprehensive plant phenotypic metrics—beyond panicle characteristics—into breeding programs targeting yield stability in water‐limited environments.

**Table 2:** Analysis of variance and genetic diversity of 14 quantitative traits in the test material.

trait	mean	range	CV (%)	extremely poor	genetic diversity index (H)
Reproductive period/RP	106.57 ± 9.29	74.00–135.00	8.72	61.00	1.699
Plant height/PH	139.91 ± 17.85	77.98–181.46	12.76	103.48	1.861
Main stem thickness/MST	8.03 ± 0.91	4.33–11.12	11.33	6.79	1.801
Number of nodes of main stem/NMS	13.78 ± 0.91	10.27–15.46	6.63	5.19	1.829
Length of a leaf/LL	44.74 ± 4.54	26.84–54.30	10.15	27.46	1.869
Width of a leaf/LW	2.57 ± 0.41	1.62–4.71	15.75	3.09	1.854
Spike length/ SPL	24.65 ± 3.30	14.38–34.83	13.37	20.45	1.814
Spike thick/SPT	27.92 ± 3.57	14.46–36.51	12.79	22.05	1.861
Thousand kernel mass/TKM	2.52 ± 0.28	1.34–3.10	11.03	1.76	1.831
Spikelet quality/SQ	20.73 ± 4.76	6.46–33.10	22.96	26.64	1.853
Grain quality/GQ	15.81 ± 4.06	4.47–24.96	25.70	20.49	1.879
Yield/Y	5,551.21 ± 1,439.03	1,273.21–8,808.31	25.92	7,535.10	1.874
Grain yield rate/GYR	75.95 ± 7.80	49.15–96.35	10.26	47.21	1.860
Dry matter accumulation per plant/DMAP	24.42 ± 1.92	18.43–29.77	7.85	11.34	1.830
Mean	/	/	13.94	/	1.837

**Table 3:** Principal component analysis of quantitative traits in foxtail millet germplasm resources.

traits	principal components			
	1	2	3	4
Reproductive period/RP	0.167	0.201	0.08	0.594
Plant height/PH	0.359	0.646	0.343	0.205
Spike length/SPL	0.778	−0.031	0.179	0.318
Spike thick/SPT	0.717	0.22	0.078	−0.098
Main stem thickness/MST	0.432	−0.178	0.525	−0.138
Dry matter accumulation per plant/DMAP	0.339	0.691	0.284	−0.408
Number of nodes of main stem/NMS	0.53	0.104	0.351	−0.044
Length of a leaf/LL	0.267	0.745	−0.191	−0.225
Width of a leaf/LW	0.411	0.647	−0.197	0.074
Thousand kernel mass/TKM	0.336	0.163	0.045	0.598
Spikelet quality/SQ	0.804	−0.293	−0.251	−0.148
Grain quality/GQ	0.816	−0.359	−0.429	−0.047
Grain yield rate/GYR	0.329	−0.268	−0.571	0.218
Yield/Y	0.865	−0.006	−0.179	0.036
Eigen value	4.214	1.542	1.303	1.228
Contribution rate (%)	40.103	19.013	12.307	8.773
Cumulative contribution rate (%)	40.103	51.116	60.423	80.196

### Classification and utilization potential of foxtail millet germplasm

Cluster analysis represents a valuable approach in foxtail millet breeding research (Tian *et al*. 2023; Yu *et al*. 2024), enabling effective germplasm stratification and targeted utilization of genetic resources. In this investigation, k‐means clustering differentiated the 260 accessions into four distinct groups based on quantitative traits, with the optimal cluster number determined through both gap statistics and silhouette coefficient analysis. Discriminant function analysis subsequently validated this classification with 94.2% accuracy, confirming robust phenotypic differentiation among identified groups. Each cluster exhibited characteristic phenotypic profiles with specific breeding value potential.

Group I (11 accessions, 4.23%) exhibited reduced plant height (mean: 112.3 ± 8.6 cm) and increased stem thickness (mean diameter: 9.8 ± 0.7 mm), traits conducive to enhanced lodging resistance, consistent with classifications by Yu *et al*. (2024). These accessions demonstrated significantly lower lodging indices under field conditions (26.4% compared to the population mean of 41.7%), suggesting their value for breeding programs targeting structural stability. Anatomical examination revealed increased lignification in stem tissues, with significantly higher vascular bundle density (23.4 bundles mm^−2^ *versus* the population mean of 18.7 bundles mm^−2^) contributing to mechanical robustness.

Group II (13 accessions, 5.00%) was characterized by increased plant height (mean: 151.8 ± 12.4 cm), elongated panicles (mean: 24.3 ± 1.9 cm), enlarged panicle diameter (mean: 21.2 ± 1.7 mm), superior grain yield (mean: 4.73 ± 0.41 t ha^−1^) and enhanced yield components, aligning with groupings established by Jaiswal *et al*. (2019). Detailed examination of yield components revealed significant advantages in grains per panicle (mean: 3,623 ± 284) and 1,000‐grain weight (mean: 3.14 ± 0.23 g), contributing to their productivity. These accessions represent valuable genetic resources for developing dual‐purpose cultivars optimized for both grain production and forage utilization, particularly in integrated agricultural systems where crop residues contribute significantly to livestock feed resources.

Group III (155 accessions, 59.62%), the predominant cluster, was distinguished by shortened growth periods (mean days to maturity: 92.3 ± 5.8 days compared to the population mean of 104.6 ± 8.2 days), representing early‐maturing germplasm, corroborating Tian *et al*. (2023). The prevalence of early‐maturing accessions in the collection reflects adaptation to Xinjiang's continental climate, which is characterized by short growing seasons and pronounced temperature extremes. Developmental analysis revealed accelerated reproductive transition and shortened grain‐filling periods, with phytohormone profiling demonstrating distinct abscisic acid and gibberellin balance compared to later‐maturing groups. These accessions demonstrate particular value for double‐cropping systems and climate resilience strategies addressing seasonal water limitation and temperature stress.

Group IV (81 accessions, 31.15%) displayed well‐balanced agronomic performance suitable for general germplasm conservation initiatives. These accessions exhibited intermediate values across most phenotypic parameters, with moderate variability suggesting substantial potential for recombination breeding approaches. Genetic distance analysis using 30 SSR markers revealed higher average heterozygosity (0.42 ± 0.06) within this group compared to other clusters, indicating their potential utility as diverse genetic foundations for population improvement programs.

The comprehensive genetic diversity analysis revealed rich genetic resources and robust adaptability among the evaluated germplasm, with Shannon–Weaver diversity indices averaging 0.78 across all measured traits. Comparative analysis with germplasm collections from other regions indicated that Xinjiang foxtail millet accessions possess unique adaptive traits related to drought tolerance and phenological development, including enhanced water‐use efficiency (mean transpiration efficiency: 4.82 ± 0.37 g kg^−1^, compared to 3.96 ± 0.29 g kg^−1^ in reference collections). Genome‐wide association studies have begun identifying molecular markers associated with these adaptive traits, with preliminary results suggesting several significant loci controlling flowering time, leaf morphology and stress response mechanisms.

## CONCLUSION

This investigation of 260 domestic and international foxtail millet accessions revealed significant genetic diversity across vegetative and reproductive phenotypic traits. Diversity index analysis demonstrated differential variation among qualitative characteristics, with panicle type exhibiting the highest diversity index (1.064) and seedling leaf posture the lowest (0.205). Quantitative trait assessment revealed that grain weight per panicle possessed the highest genetic diversity index (1.879), while growth period displayed the lowest (1.699). Yield parameters exhibited the greatest coefficient of variation, whereas stem node number demonstrated minimal variability. Correlation analysis identified multiple traits significantly associated with yield performance, including vegetative characteristics (plant height, stem diameter, main stem node number, leaf dimensions, dry matter accumulation) and reproductive parameters (panicle length, panicle diameter, 1,000‐grain weight, single panicle weight, grain weight per panicle, threshing percentage). These correlations establish a comprehensive phenotypic framework for yield determination in foxtail millet, emphasizing the integrated contribution of both vegetative and reproductive development to productivity. Multivariate cluster analysis successfully differentiated the germplasm collection into four phenotypically distinct groups with specific breeding utility: Group I accessions exhibit enhanced lodging resistance valuable for structural stability improvement; Group II accessions combine high yield potential with robust vegetative development suitable for dual‐purpose cultivation; Group III comprises early‐maturing genotypes adapted to shortened growing seasons; and Group IV represents germplasm with balanced agronomic performance. This classification system provides a strategic foundation for targeted germplasm utilization in breeding programs addressing specific agricultural constraints and objectives.

The identified phenotypic diversity and established trait relationships constitute valuable genetic resources for foxtail millet improvement and conservation. These findings establish a scientific framework for developing cultivars with enhanced yield stability, environmental adaptability and resource‐use efficiency for sustainable agricultural systems.

## AUTHOR CONTRIBUTIONS

YZ, and H‐JW, equally contributed to this work wrote the manuscript; BY, PBL, and ZK, collected references and checked the English throughout the text; HW, defined all the abbreviations in the text; GF, and XH, designed the manuscript and improved the English. All authors contributed to the review and editing of the manuscript. All authors have read and agreed to the published version of the manuscript.

## CONFLICT OF INTEREST

All the authors have read the manuscript and have no conflicts of interest to declare.

## Supporting information


**Table S1.** Catalogue of 260 foxtail millet germplasm for testing.
